# The Johns Hopkins Hospital Reports

**Published:** 1900-03

**Authors:** 


THE JOHNS HOPKINS HOSPITAL REPORTS. Volume VII., Nos. 5-6-7 8-9. Hernia. by JOSEPH C. BLOODGOOD, M. D. Baltimore: The Johns Hopkins Press. 1899.

This volume contains the report of 459 operations for hernia with special consideration of 268 cases which were treated by the Halsted method, and also of the transplantation of the rectus muscle in certain cases of inguinal hernia in which the conjoined tendon was found obliterated. It is a magnificent treatise on the subject which reflects great credit upon the surgical skill of Halsted and his assistants and their accuracy in recording the histories and final results of their cases. There is no branch of the subject, from the practical or rather surgical aspect, which has not been thoroughly discussed.

The excision of the veins of the cord to reduce its bulk and further guard against recurrence; the use of silver wire and foil; the effect of rubber gloves in reducing the percentage of suppurating cases to 1.8 per cent.; a new operation for varicocele, castration and hydrocele by excision in the groin; the transplantation of the rectus muscles in cases where the palpable portion of the conjoined tendon is obliterated; atrophy of testes; operation under cocaine anesthesia; the study of the ultimate results in which the two chief causes of recurrence are found to be obliteration of the conjoined tendon and transplantation in toto of the cord, without excision of the veins in the upper angle of the wound; the acute infections and other complications following operation; and the ultimate health of the patient, are among the many and varied aspects of the operative treatment of hernia which are so carefully and scientifically treated in this volume.

It would far exceed the limits of space allowable to adequately review this work. The illustrations are admirable in clearness. In fact, a surgeon familiar with the anatomy of the inguinal region could closely imitate the Halsted operation by a study of the plates alone. It is a valuable record which every surgeon should possess and familiarise himself with. J. P.

